# Diallyl Trisulfide, a Biologically Active Component of Garlic Essential Oil, Decreases Male Fertility in *Sitotroga cerealella* by Impairing Dimorphic Spermatogenesis, Sperm Motility and Lipid Homeostasis

**DOI:** 10.3390/cells12040669

**Published:** 2023-02-20

**Authors:** Sakhawat Shah, Karam Khamis Elgizawy, Chun-Mei Shi, Hucheng Yao, Wen-Han Yan, Yu Li, Xiao-Ping Wang, Gang Wu, Feng-Lian Yang

**Affiliations:** 1 Hubei Key Laboratory of Insect Resources Utilization and Sustainable Pest Management, College of Plant Science and Technology, Huazhong Agricultural University, Wuhan 430070, China; 2 Plant Protection Department, Faculty of Agriculture, Benha University, Moshtohor, Toukh 13736, Egypt; 3 College of Horticulture and Forestry, Huazhong Agricultural University, Wuhan 430070, China; 4 College of Informatics, Huazhong Agricultural University, Wuhan 430070, China

**Keywords:** diallyl trisulfide, dimorphic spermatogenesis, sperm motility, male infertility, *Sitotroga cerealella*

## Abstract

Diallyl trisulfide (DAT) is a biologically active component of garlic essential oil and exhibits multi-targeted activity against many organisms. The current study tested the capacity of DAT to decrease the male fertility of *Sitotroga cerealella*. The effects on testis morphology, sperm number, motility, and lipid homeostasis were observed in adult males fumigated with DAT at a dose of 0.01 μL/L in air. The results indicated that the DAT significantly decreased the dimorphic sperm number. Meanwhile, the ultrastructural analysis of the sperm showed that the DAT caused malformed and aberrant structures of mitochondrial derivatives of dimorphic sperm. Additionally, the lipid homeostasis and ATP contents in the male adults were significantly decreased after treatment. Moreover, the total sperm motility was reduced, while the wave-propagation velocity, amplitude, frequency, and wavelength were significantly decreased compared with the controls. Overall, this study reported, for the first time, that DAT impairs energy metabolism, inhibits dimorphic spermatogenesis, and decreases sperm motility, while these abnormalities in sperm lead to adult-male infertility.

## 1. Introduction

When fresh garlic is chopped or crushed, alliin, via alliinase, is converted into allicin. The allicin generated is unstable and quickly hydrolyzed into a series of other sulfur-containing compounds, such as diallyl disulfide, diallyl trisulfide (DAT), and diallyl tetra sulfide [1]. Diallyl trisulfide has the highest representation (45%) among the organosulfur compounds in garlic and has potential pharmacological functionalities against several physiological processes [2]. It shows anticancer activity in human colon cancer through microtubule-network-formation disruption, apoptosis induction, and mitotic arrest [3]. Several studies reported that DAT inhibits the initiation of tumorigenesis and suppresses cell growth, inducing apoptosis in lung-, prostate- and multiple-cancer cells [3]. Diallyl trisulfide is believed to be a potent source of hydrogen sulfide (H2S) and reduces the risk of ischemic myocardium and ethanol-induced fatty liver through an H2S-mediated mechanism [4].

*Sitotroga cerealella* Olivier (Lepidoptera, Gelechiidae) is a worldwide destructive pest of staple crops. Recent studies reported that DAT decreases the oviposition of adult *S. cerealella* [5] and suppresses egg hatching and fertility in stored-product pests [6]. However, the reason behind this ovipositional reduction remains unclear. We hypothesize that DAT may cause disruption in male moth spermatogenesis and females may receive fewer viable sperm from their treated male partners, which causes a reduction in the oviposition of moths.

Several phyla, such as Mollusca, Arthropoda, Chordata and Rotifera exhibit dimorphic spermatogenesis [7,8,9]. In insecta, Lepidopteran species possess dichotomous spermatogenesis. Similar to other lepidopteran moths, *S. cerealella* possesses dimorphic sperm that differ in structure and function. Eupyrene sperm possess a nucleus and DNA, which fertilize eggs while the apyrene sperm lack a nucleus and nuclear DNA and play a role in the transfer of eupyrene sperm to the female reproductive organ [10,11]. Spermatogenesis in the testis of moths begins in the larval stage and continues into the adult stage. The moths at the larval stage possess two lobes of testis, which fuse in the late pupal stage. Mature sperm move from the testis to the seminal vesicles and are then transferred from the male to the female during insemination. The sperm undergo several post-ejaculatory modifications and acquire motility when mixed with accessory-gland secretions in the spermatophore [12].

The present study aims to evaluate the testis morphology, sperm number, sperm motility, and lipid homeostasis in adult males fumigated with DAT, compared to those in the testes of normal unfumigated adult males. Moreover, the adenosine triphosphate (ATP) content was also evaluated.

## 2. Materials and Methods

### 2.1. Tested Insects and DAT Fumigation

The strain of *S. cerealella* associated with wheat was maintained in a laboratory at 28 °C ± 1 °C and 75% ± 5% relative humidity, with a photoperiod of 14:10 h (light: dark). This strain, from Wuhan, Hubei province, China, was used for experimental tests with DAT (purity > 90%, Sigma-Aldrich, Steinheim, Switzerland). Virgin adult moths were obtained from individual grain containing pupae placed in small glass tubes capped with cotton cloths and held at the laboratory as described above. Pupa were inspected hourly for adult emergence, after which one-day-old adult males were collected for experiments. Ten adult males were treated by fumigation with DAT at a dose of 0.010 μL/L in glass pots (1000 mL, 10 cm diameter × 13 cm height) for 7 h, as previously described [13]. Adult male moths fumigated with DAT at a dose of 0.010 μL/L had normal mating behavior and successful insemination. After treatment, adult males were collected and processed for subsequent experiments accordingly. Each experiment consisted of two groups with three replicates from control (CK) and treated (DAT) moths.

### 2.2. Transmission Electron Microscopy of Testes

Testes of 15 adults were isolated and fixed in 2.5% glutaraldehyde and 4% paraformaldehyde in 0.1 M of phosphate buffer (pH 7.3) for 24 h at room temperature and post-fixed in 1% osmium tetroxide in the same buffer for 2 h. Samples were then washed in distilled water, stained with an aqueous solution of 0.5% uranyl acetate for 2 h, dehydrated in a series of increasing concentrations of acetone (50%, 70%, 90%, and 100%), and embedded in resin (Araldite^®^). Ultrathin sections were stained in a saturated alcoholic solution of uranyl acetate and lead citrate and analyzed using a 1200E TEM (JEOL, Tokyo, Japan). Images were captured with a MORADA-G2 TEM camera (Olympus, Tokyo, Japan).

### 2.3. Determination of Lipid Droplets

Lipid droplets were visualized by staining fat bodies with Nile red (Sangon Biotech, Shanghai, China). Dissected fat bodies from adult male moths were washed three times in 1 × phosphate-buffered saline (PBS), and then fixed in 4% paraformaldehyde for 30 min at room temperature. After washing three times with PBS containing 0.1% Triton X-100 (PBST), no lipid was removed in this step. The tissues were incubated in 0.1% PBST buffer with 1 μg/mL Nile red solution for 90 min at room temperature. Tissues were then incubated for 15 min in 1 μg/mL 4′,6-diamidino-2-phenylindole (DAPI; Thermo Fisher Scientific, Waltham, MA, USA) at RT followed by washing with 0.1% PBST three times to stain nuclei. Finally, tissues were washed twice with 1 × PBS and transferred to microslides. Slides were observed under a fluorescent microscope BX51 (Olympus, Tokyo, Japan).

### 2.4. Morphological Observation and Quantity Statistics of Sperm

For sperm morphological and quantification analysis, seminal vesicles from adult male *S. cerealella* were dissected and washed in PBS. The seminal vesicles were poked with a pin, and the contents were separated with tweezers and distributed evenly into PBS buffer and placed in 1.5-milliliter centrifuge tubes [14]. Each tube contained 5 seminal vesicles. The samples were shaken and PBS was added up to 80 µL, followed by addition of 20 µL cold DAPI to and staining of the samples at Line 111. The RT was placed between brackets, preceded by full name of abbreviation, i.e., retention time (RT) in dark environment for 10–15 min. Next, 2 µL of sample were pipetted and placed on microscopic slide on 10 rectangular places, each with a dimension of 2 mm × 3 mm.

The total number of spermatozoa were counted with formula [15]:

(1) Sperm in seminal vesicles=((24×24)/(10×2×3)×A×50)/3

where A = sperm number in 10 rectangles.

### 2.5. Sperm-Survival Assay

The seminal vesicles from adult male *S. cerealella* were dissected and washed in PBS. The seminal vesicles were pierced with a needle and the spermatozoa were collected. The contents were distributed gently in PBS and placed in 1.5-milliliter centrifuged tube. Each tube contained 5 seminal vesicles. The samples were shaken and again PBS was added up to 500 µL along with 5 µL SYBR Green I. The samples were then incubated for 5–10 min at 36 °C. After incubation, 5 µL propidium iodide (PI) was added, mixed thoroughly and placed at RT in dark environment for 5–10 min. Next, we pipetted 2 µL sample and placedon microscopic slide on 10 rectangular places, each with a dimension of 2 mm × 3 mm. The samples were then observed under the inverted fluorescent OLYMPUS IX71 microscope. Each group was counted three times, with 100 sperm each, i.e., a total of 300 sperm were counted.

The sperm-survival rate was calculated with formula:

(2) Sperm survival rate (%)=(alive sperm)/(total number of sperm)×100

### 2.6. Determination of Total Sperm Motility

Total sperm motility of spermatophore in bursa copulatrix of female moths mated with DAT-treated males and control was determined with computer-assisted sperm-motility analyzer (CASA), Hamilton thorn CEROS II. Briefly, control and DAT-fumigated male moths were mated with female partners in a separate glass vial. Upon completion of copulation, the individual females from each glass vial were separated and the spermatophore in bursa copulatrix were dissected under stereomicroscope. The tissues were punctured with a needle in a PBS drop. Sperms were placed in the viewing chamber and live video pictures were visualized using a setup comprising an Olympus BX40 microscope (Olympus Optical Co., Tokyo, Japan), a 10-times-negative-phase objective and a Basler digital camera (model acA1920-155 Basler AG, Vision Technologies, Ahrensburg, Germany). Sperm motility was estimated subjectively by a third-person observer in a blinded manner. Sperm were classified as motile if they presented any type of active movement. Motility was scored on a scale of 0 to 5, corresponding to 0, 20%, 40%, 60%, 80% and >95% of the observed population being motile.

### 2.7. Motile-Sperm-Image Acquisition, Processing and Measurement

Sperm flagellar wavelength, velocity, amplitude and frequency were measured using a Nikon Ti Microscope at 10× or 20× magnification. NIS-Elements Viewer software and a color camera, Nikon DS-Ri2, were used for recording. All images were processed with image-processing software, ImageJ, FIJI and plugin tools. The detailed methodology for image processing and parameter analysis followed that presented by De Los Santos (2015) [16], with minor modifications. Wave-propagation velocity was calculated by measuring the speed of undulating waves by clicking on the peak of the wave in each frame using Manual Tracking Plugin in ImageJ. We then averaged the instantaneous velocity for each wave. The amplitude was measured from an approximate midpoint in middle of wave from middle peak to trough with the line tool at 0 degrees. The wavelength was measured using a line tool from peak to peak or trough to trough, depending on what was shown on the wave and which was easiest to measure. The frequency was measured by examining the stack, choosing a frame and then examining the stack again to identify where the waveform repeated through a visual estimation of a complete cycle.

### 2.8. Determination of Adenosine Triphosphate (ATP) Contents in Sperm

The ATP concentration was determined using a luciferase-based ATP bioluminescence Assay ATPlite kit (Perkin Elmer, Waltham, MA, USA), according to the manufacturer’s instructions. Briefly, 50 µL of mammalian-cell-lysis solution was added to 100 µL of sperm-cell suspension taken from seminal vesicles of control and DAT-fumigated adult males per well of microplate. Next, 50 µL of substrate solution was added to each well, shaken and dark-adopted. The luminescence was measured with Envision multimode plate reader (Perkin Elmer USA). The experiment was performed in triplicates with biological replicates and a total of 50 male moths from each group were used.

### 2.9. Measurement of Triacylglycerol and Cholesterol Contents

Fat bodies were dissected from control and DAT-fumigated adult males. Tissues were washed three times in 1× PBS and homogenized separately in lysis buffer. Lysates were maintained at room temperature for 10 min, after which they were heated for 10 min at 70 °C and centrifuged for 5 min at 2000× *g* at RT. Triglyceride and cholesterol contents of supernatants were measured using a Tissue Triglyceride and Cholesterol Assay Kit (Nanjing Jiancheng Biological Reagent, Nanjing, China). Protein concentrations were analyzed using a Bicinchoninic Acid (BCA) Protein Assay Kit (Thermo Fisher Scientific), according to the manufacturer’s instructions.

### 2.10. Statistical Analysis

The data were analyzed using Student’s t test. All the figures were generated using GraphPad Prism 8 (GraphPad Software Inc., San Diego, CA, USA). The data were presented as the mean  ±  standard error mean (mean  ±  SEM). Statistical tests used to determine significance are indicated in figure legends.

## 3. Results

### 3.1. DAT Reduced the Number and Survival of S. cerealella Spermatozoa

**Figure 1:**
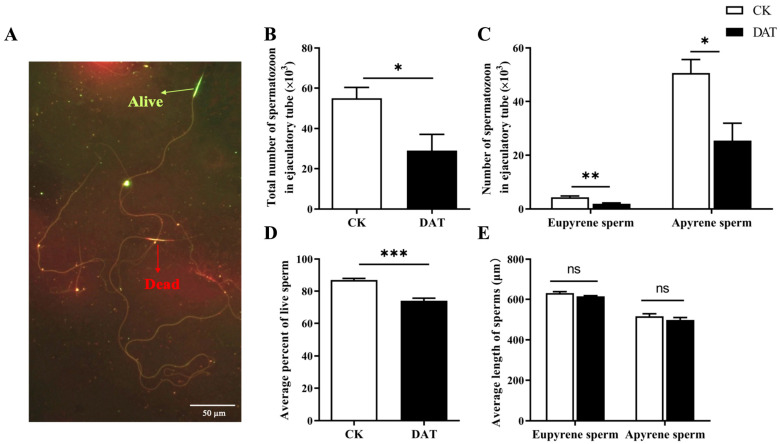
Quantification, survival and motility analysis of *S. cerealella* spermatozoa. (**A**) Representative images of viable sperm from adult seminal vesicles, stained with SYBR. Green marks live and red marks dead sperm. (**B**–**E**) Sperm-quantification assays. Three replicates and each replicate with 5 seminal vesicles were taken for each test (unpaired two-tailed *t*-test, *** *p* < 0.001, ** *p* < 0.01, * *p* < 0.05, ns = non-significant). Error bars depict standard error mean (SEM).

We quantified total sperm number, sperm length, and sperm survival for control and DAT-treated male moths. The results showed that spermatozoa counts were significantly reduced after DAT treatment (*p* < 0.05, df = 10, F = 1.133 Figure 1B). The DAT significantly reduced the sperm number for both apyrene (*p* < 0.05) and eupyrene sperm (*p* < 0.01; df = 10, F = 1.086; Figure 1C). The DAT exposure reduced the percentage of live cells (Figure 1A) to 74% ± 1.50%, which was significantly less than the control (87% ± 1.00%) (*p* < 0.001, df = 6, F = 0.99; Figure 1D). However, no adverse effects of the DAT were recorded along the lengths of the apyrene and eupyrene spermatozoa (*p* = 0.323; df = 48, F = 0.043; Figure 1E).

### 3.2. DAT Altered Ultrastructure of Apyrene and Eupyrene Sperm of S. cerealella

**Figure 2:**
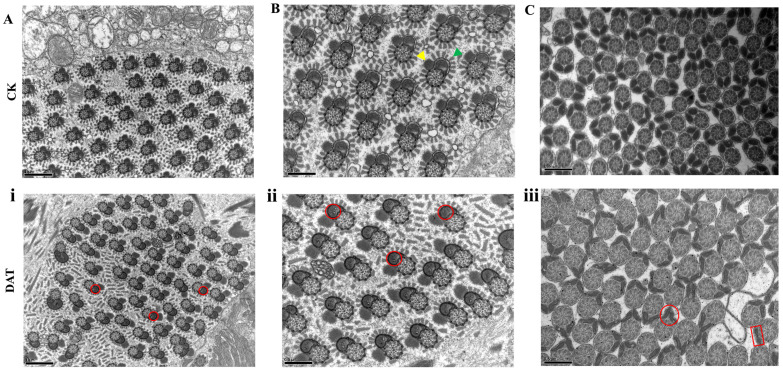
Transmission electron microscopy of *S. cerealella* apyrene and eupyrene sperm cells. (**A**,**B**) Transverse sections of the anterior region of eupyrene sperm bundles in testes of control moths. Yellow arrowhead indicates reticular appendage and green arrowhead shows lacinate appendage enlargement of (**i**,**ii**) cross-section of eupyrene sperm in testes of DAT-fumigated moth containing aberrant lines and malformed mitochondria (red circles). (**C**) Transverse sections of apyrene sperm bundles in testes of control adults. Control apyrene sperm show normal morphology of two mitochondrial derivatives of apyrene sperm. The two mitochondrial derivatives indicate electron lucent end paracrystalline core and accessory tubules. (**iii**) Transverse section of apyrene spermatozoa in testes of DAT-fumigated adult moths. Red rectangular box and circle indicate abnormal and shrunken mitochondrial derivatives. The one end of mitochondrial derivatives indicates narrow peaks. The scale bars are shown in the lower left of the images. The scale bar for Figure **A**,**i** = 1 um and **B**,**ii**,**C**,**iii** = 0.5 um.

The changes in the ultrastructures of the eupyrene and apyrene spermatozoa were observed through a TEM of both the control and the DAT-fumigated groups. The control groups showed axoneme and mitochondrial derivatives of dimorphic sperm with normal morphologies (Figure 2A–C). The eupyrene sperm structure of the DAT-fumigated moths showed malformed mitochondrial derivatives with aberrant lines (Figure 2i,ii). The mitochondrial derivatives of the eupyrene sperm also exhibited morphological variation compared with the controls; however, no significant defects were found in the axoneme structure and a normal microtubule distribution was observed. The morphologies of the apyrene sperm from the DAT-fumigated moths were different from those in the control groups. The control groups showed two turgor mitochondrial derivatives of sizes equal to those of the axoneme; however, mitochondrial derivatives with shrunken and abnormal sizes were observed in the treated groups (Figure 2iii). Altogether, these results allow us to conclude that DAT leads to defective and malformed mitochondrial derivatives in mature dimorphic sperm, which has vital roles in flagellar beating and sperm motility.

### 3.3. DAT Decreased the Sperm Motility of S. cerealella

**Figure 3:**
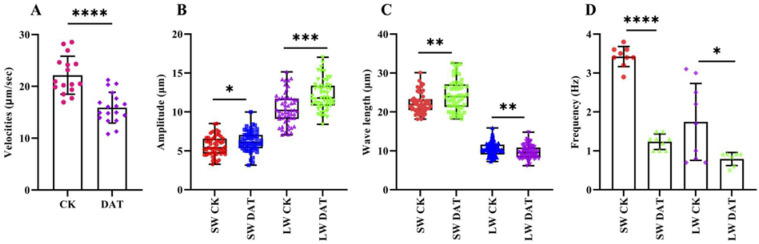
Female reproductive tracts of females mated with control or fumigated moths were dissected and the sperm were analyzed under a phase-contrast microscope. The videos were recorded at 17 fps and then analyzed on ImageJ for calculation of different parameters, as discussed in Methods. Velocities (**A**), amplitude (**B**), wavelength (**C**) and frequencies (**D**) were counted for all samples (unpaired two-tailed *t*-test, **** *p* < 0.0001, *** *p* < 0.001, ** *p* < 0.01, * *p* < 0.05).

Spermatophore in bursa copulatrix of female moths mated with DAT-treated males or controls were isolated and visualized, and the sperm movement was quantified on a scale of 0 to 5. In total, 50 FRTs from each group were dissected and the movement of the sperm was observed. The sperm from the females mated with the control moths exhibited active and hyper-motile flagellar vibration (Movie S1), while immobility and weak movement of sperm were recorded in the reproductive tracts of the females mated with the DAT-fumigated moths (Movie S2). The control groups showed 80 to 95% percent motile cells; however, only two samples from the DAT-treated groups possessed 60% motility (Table 1). These findings clearly suggest that the DAT significantly reduced the sperm motility. The sperm exhibited two types of waves, which we named “large wave” (LW) and “small wave” (SW), respectively (Movies S1 and S2). The waves originated behind the head, moved toward the tail and propelled the sperm through the medium. A double helical movement of the wave was observed somewhat clockwise with a mean wave-propagation velocity of 22.15 μm/s (Figure 3A). The DAT significantly reduced the instantaneous velocities of the waves. The mean wave-propagation velocities for the DAT group were recorded as 15.89 μm/s, which was significantly lower than those in the controls (*p* < 0.0001).

The LW of the sperm exhibited high amplitude, while the SW exhibited short amplitude (Figure 3B). The mean amplitudes for the SW and LW of the control groups were 5.6 μm and 10.42 μm, respectively. The DAT significantly increased the amplitudes of both waveforms. Values of 6.26 μm (*p* < 0.05) and 12.2 μm (*p* < 0.001) were recorded for the amplitudes of the SW and LW waveforms, respectively, for the DAT-treated groups.

The wavelengths of the LW and SW were recorded for the control and DAT groups and, similar to the amplitude, the LW exhibited larger wavelengths and vice versa. An increase in wavelength for the LW was recorded in the DAT groups; however, the DAT decreased the wavelength of the SW compared to the control groups (*p* < 0.01; Figure 3C). We believe that SW is responsible for the actual movement of spermatozoa; in addition, the DAT significantly decreased the wavelength of the SW, which showed that DAT negatively affects the motility of spermatozoa, as shown in the percentage of motility reduction.

We also examined the frequencies of the LW and SW for both groups. The results indicated that the SW exhibited higher frequencies than the LW, as the SW repeated itself more than the LW. The DAT significantly reduced the frequencies of both waveforms (Figure 3D). The reduction in the frequencies of both waveforms was in accordance with the low motility of the DAT-group sperm.

### 3.4. DAT Decreased Sperm ATP Contents of S. cerealella

**Figure 4:**
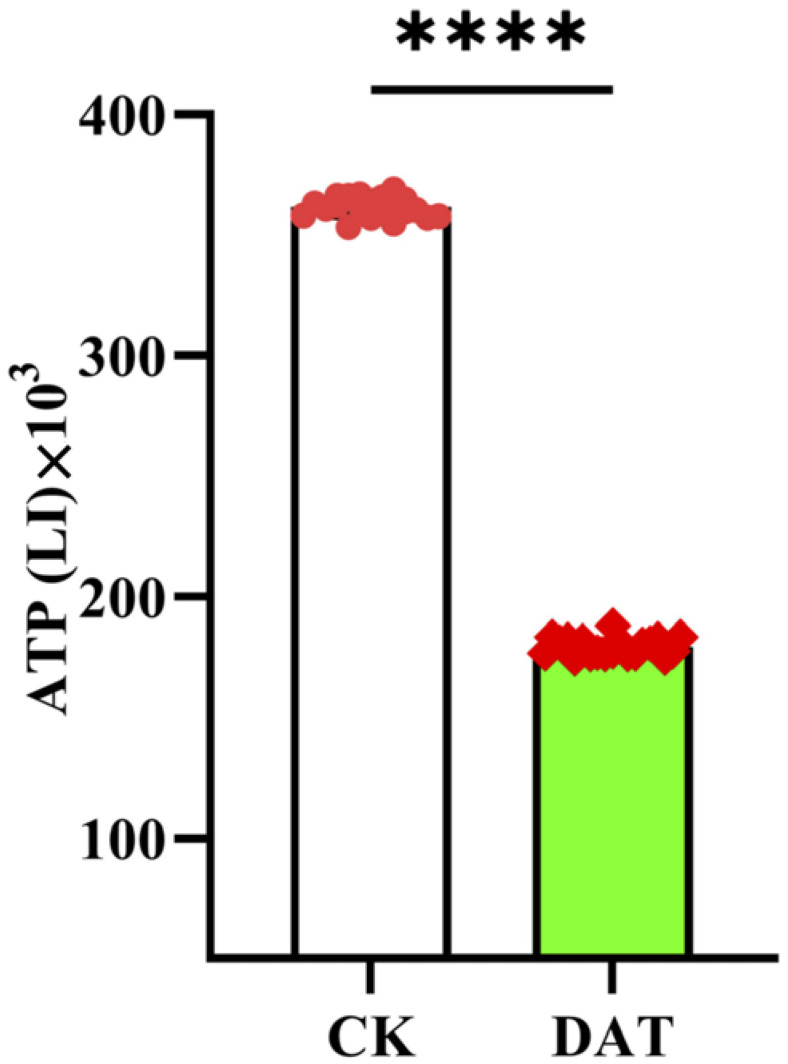
Rate of adenosine triphosphate (ATP) was measured in spermatozoa from seminal vesicles of control and DAT-fumigated male moths. The experiment was performed in triplicates with biological replicates and used a total of 50 male moths from each group. The data are represented as luminescence intensity (LI) (unpaired two-tailed *t*-test, **** *p* < 0.0001).

For sperm motility and metabolic activities, Adenosine triphosphate (ATP) is considered the main energy source. Given the reduction in sperm motility after the DAT fumigation, we measured the ATP luminescence in the control and DAT-fumigated-moth spermatozoa. The sperm-cell suspensions from both groups were processed for ATP measurement using a luciferase-based ATP bioluminescence assay kit. The results revealed that the DAT reduced the ATP contents compared with the controls (Figure 4; *p* < 0.0001). These results are in accordance with those of the sperm-motility analysis.

### 3.5. DAT Decreased Lipid Homeostasis of S. cerealella

**Figure 5:**
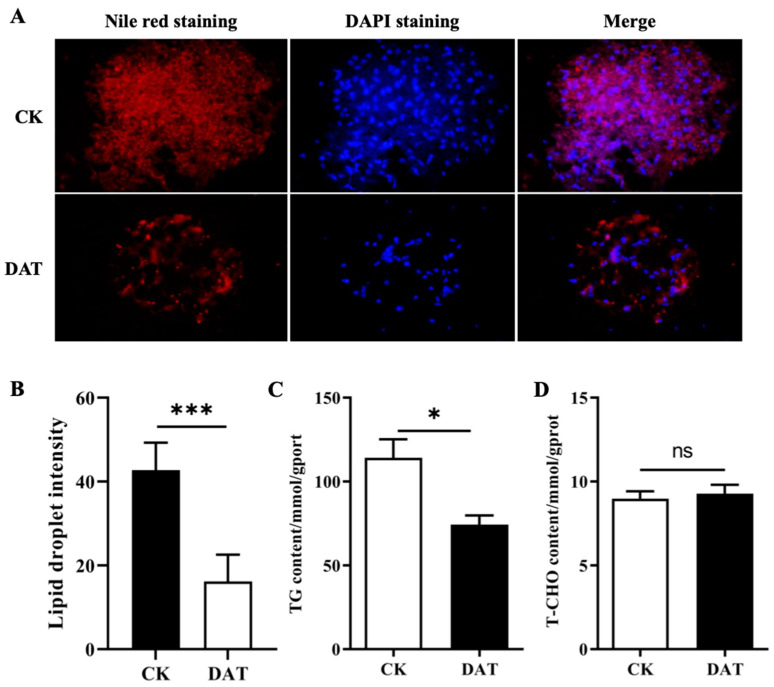
Lipid droplets and triacylglycerol characterization. (**A**,**B**) Characterization of lipid droplets in the fat bodies of control and DAT-fumigated adult male moths. Fat bodies of adult male moths were dissected and stained by Nile red and nuclei in the fat bodies were stained by 40,6-diamidino-2-phenylindole (DAPI), shown as red and blue fluorescence. The (CK) marks the control group, showing strong fluorescence of lipid droplets. Tight arrangement of lipid droplets’ grooves-like structures are evident in fat bodies. The (DAT) indicates the lipid droplets in the fat bodies from DAT-fumigated moths. Weak fluorescence, empty spaces and small sizes indicated. (**C**) Determination of triacylglycerol content from the fat bodies of male adult moths of control and DAT-fumigated groups. The DAT fumigation significantly reduced the triacylglycerol contents in the adult-male-moth fat bodies. (**D**) Total cholesterol content from the fat bodies of male adult moths from control and DAT-fumigated groups. Control and DAT groups showed no significant difference in the quantification of cholesterol contents. Triacylglycerol contents (mmol triglyceride g − 1 total protein) and cholesterol content (mmol cholesterol g − 1 total protein) were determined from three independent biological replicates (mean ± standard deviation; *n* = 5). CK (control group), DAT (DAT-fumigated adult moths). Figure data expressed as mean ± SE; ns (non-significant difference), * *p* < 0.05 *** *p* < 0.001 (*t*-test).

Lipid and cholesterol homeostasis are vital for male fertility and reflect sperm concentration and motility. Because the DAT significantly decreased the sperm motility and related parameters, we examined the lipid contents in the fat bodies of the control and DAT-fumigated moths using a fluorescence assay. The fat-body particles in the control moths were tightly arranged and large (Figure 5A, CK); however, after the DAT exposure, the fat bodies had weak fluorescence and empty spaces, with small fat-body particles (*p* < 0.001; Figure 5A, DAT panel and B), which means that the lipid contents decreased after treatment (Figure 5B). We further checked for triacylglycerol and total cholesterol content in the controls and the DAT-treated male moths (Figure 5C,D). The results showed that a reduction in the content of triacylglycerol was found in the DAT-treated males and the difference was significant compared with the controls (*p* < 0.05, df = 4, F = 2.489). However, no significant changes were seen in the total cholesterol content between the controls and the treated groups (Figure 5D).

## 4. Discussion

Garlic belongs to the Amaryllidaceae family, which produces allicin, diallyl disulfide, diallyl trisulfide and other organosulfur compounds. The consumption of garlic has been associated with the prevention of many cancers and cardiovascular diseases [17,18]. At a dose of 0.01 μL/L, DAT, a highly bioactive component from garlic essential oil, inhibits oviposition, despite normal mating behavior [19]. To better determine the reduction in oviposition, this study showed that DAT caused a reduction in the total sperm counts of male moths accompanied by a decrease in viable sperm. Furthermore, the sperm transferred to female reproductive tracts were much less motile after the female mated with a male partner exposed to DAT. Furthermore, several key parameters of sperm motility were also significantly disrupted by the DAT fumigation. We observed a complex flagellar movement in *S. cerealella* sperm, consisting of two waveforms: a wave of small amplitude with higher frequency, which was simultaneously overlaid on a wave with a higher amplitude and smaller frequency. We named these small wave (“SW”) and large wave (“LW”), respectively. Similar waveforms have been observed in the sperm flagellar movements of other insect species, such as mosquitoes and beetles, which were named small and large waves, while some studies distinguished them as “major” and “minor” waves [16,20]. The function and formation of these waveforms are paradoxical [20]. It was suggested that small waves are responsible for the actual movement of sperm, whereas the large waves form as a result of the static force of the axoneme [20]. We propose that the movement of both waves is vital for the propagation of dimorphic sperm in FRT; hence, DAT affects the magnitude of both waveforms and decreases sperm movement and relative parameters.

Moreover, the ATP contents were significantly reduced by the DAT, which is the main energy source for sperm motility and metabolic activities. A reduction in ATP is associated with lower motility [21]. The DAT negatively affects spermatogenesis in *S. cerealella* testes. After DAT exposure, eupyrene sperm showed malformed and abnormal mitochondrial structures. Similar observations were recorded in apyrene sperm cells, in which the mitochondrial derivatives changed morphologically and displayed a shrunken structure. The function of eupyrene-sperm mitochondrial derivatives is still unclear; however, several roles were suggested for apyrene mitochondrial derivatives, such as a passive role in sperm motility, the production of energy, and the activation and nourishment of oocytes [20]. The literature suggests that abnormal mitochondrial derivatives of apyrene sperm in *Bombyx mori* lead to a complete loss of sperm motility [10], which implies the importance of normal apyrene-sperm structures in the fertilization of lepidopteran moths. We witnessed that the DAT caused a decrease in sperm motility and relative parameters, while abnormal mitochondrial derivatives were seen in both the apyrene and the eupyrene sperm. We propose that DAT might cause disruption in the gene transcripts responsible for the metabolism and maintenance of sperm ultrastructure, specifically for mitochondrial derivatives. The functional inhibition of these genes by DAT may lead to abnormal morphology in mitochondrial derivatives, which eventually leads to spermatogenesis arrest and low sperm motility in the female reproductive tract and may cause ovipositional inhibition. However, subsequent experiments on the testis transcriptome will be needed for the functional characterization of the target genes manipulated by DAT.

Lipid metabolism is essential for growth, embryo development, flight, hibernation, starvation, migration and reproduction in insects [22]. Lipids are stored in adipocyte cytoplasm, in compartments known as lipid droplets (LDs). Lipids comprise neutral lipids, triacylglycerols (TAG), surrounded by a monolayer consisting mainly of phospholipids [22]. During extended nonfeeding periods or starvation, TAG is hydrolyzed into glycerol and free fatty acids in a process called lipolysis. This response is activated in response to energy demand and supplies energy substrates to other organs [23]. The lipolytic enzymes, HSL and TGL, are thought to hydrolyze stored TAG. Here, it was shown that the DAT fumigation significantly reduced the TAG content, which is the major energy reservoir in insects. Adult *S. cerealella* do not feed and reproduction entirely depends on stored energy, which is available in the form of lipid droplets and triacylglycerol contents. The fluorescence assay indicated that the DAT reduced the lipid droplets in the fat bodies of the adult moths and lowered the TAG content. A reduction in lipid metabolism and TAG is likely to be associated with sperm-motility decline and disruption to several reproductive processes. Furthermore, DAT causes defects in spermatogenesis and reduces both apyrene and eupyrene gametes in adult male *S. cerealella*. The abnormalities in sperm physiology and motility due to DAT may cause a reduction in oviposition. Transcriptome and proteome analyses of *Drosophila* and honeybee *Apis mellifera* sperm-storage organs and their secretions identified carbohydrate and lipid metabolism pathways that act to nourish sperm and regulate sperm activation and storage, as well as several other reproductive processes [24,25]. Repeated cell divisions during spermatogenesis entail the synthesis of large amounts of RNA and DNA. Therefore, the impaired spermatogenesis in DAT-fumigated male moths might reflect the impaired synthesis of these nucleic acids. Moreover, decreased testicular RNA synthesis may reflect a greater increase in an arginine-rich histone, which forms a complex with DNA, preventing it from acting as a primer for RNA synthesis [26,27,28].

## 5. Conclusions

The data revealed that DAT adversely affects dimorphic spermatogenesis and negatively regulates the fertility of *S. cerealella*. The DAT can be used in integrated pest management (IPM) programs for the suppression of pest populations, specifically for stored-product pests, via the fumigation of grains or storage houses. Further research would identify the molecular target of DAT responsible for spermatogenesis defects.

## Figures and Tables

**Table 1:** Total-sperm-motility analysis using CASA system.

% Motile Sperm	0%	20%	40%	60%	80%	95%
Scale	0	1	2	3	4	5
CK	-	-	-	-	8	42
DAT	10	25	13	2	-	-

## Data Availability

Not applicable.

## Supplementary Materials

The following supporting information can be downloaded at: https://www.mdpi.com/article/10.3390/cells12040669/s1, Movie S1: CK sperm motility and Movie S2: DAT sperm motility.

## References

[1] Ilić D.P., Nikolić V.D., Nikolić L.B., Stanković M.Z., Stanojević L.P., Cakić M.D. (2011). Allicin and related compounds: Biosynthesis, synthesis and pharmacological activity. Facta Univ. Phys. Chem. Technol..

[2] Thuy B.T.P., My T.T.A., Hai N.T.T., Hieu L.T., Hoa T.T., Thi Phuong Loan H., Triet N.T., Van Anh T.T., Quy P.T., Van Tat P. (2020). Investigation into SARS-CoV-2 resistance of compounds in garlic essential oil. ACS Omega.

[3] Seki T., Hosono T., Hosono-Fukao T., Inada K., Tanaka R., Ogihara J., Ariga T. (2008). Anticancer effects of diallyl trisulfide derived from garlic. Asia Pac. J. Clin. Nutr..

[4] Predmore B.L., Kondo K., Bhushan S., Zlatopolsky M.A., King A.L., Aragon J.P., Grinsfelder D.B., Condit M.E., Lefer D.J. (2012). The polysulfide diallyl trisulfide protects the ischemic myocardium by preservation of endogenous hydrogen sulfide and increasing nitric oxide bioavailability. Am. J. Physiol. Circ. Physiol..

[5] Chang M.M., Shah S., Wu M.Y., Zhang S.S., Wu G., Yang F.L. (2020). Effect of diallyl trisulfide on the reproductive behavior of the grain moth, *Sitotroga cerealella* (Lepidoptera: Gelechiidae). Insects.

[6] Huang Y., Chen S.X., Ho S.H. (2000). Bioactivities of methyl allyl disulfide and diallyl trisulfide from essential oil of garlic to two species of stored-product pests, *Sitophilus zeamais* (Coleoptera: Curculionidae) and *Tribolium castaneum* (Coleoptera: Tenebrionidae). J. Econ. Entomol..

[7] Hodgson A.N. (1997). Paraspermatogenesis in gastropod molluscs. Invertebr. Reprod. Dev..

[8] Koehler J.K., Birky C.W. (1966). An electron microscope study of the dimorphic spermatozoa of Asplanchna (Rotifera). Z. Für Zellforsch. Und Mikrosk. Anat..

[9] Alberti G. (2005). Double spermatogenesis in Chelicerata. J. Morphol..

[10] Chen S., Liu Y., Yang X., Liu Z., Luo X., Xu J., Huang Y. (2020). Dysfunction of dimorphic sperm impairs male fertility in the silkworm. Cell Discov..

[11] Sakai H., Oshima H., Yuri K., Gotoh H., Daimon T., Yaginuma T., Sahara K., Niimi T. (2019). Dimorphic sperm formation by Sex-lethal. Proc. Natl. Acad. Sci. USA.

[12] Pitnick S., Wolfner M.F., Dorus S. (2020). Post-ejaculatory modifications to sperm (PEMS). Biol. Rev..

[13] Shah S., Hafeez M., Wu M.-Y., Zhang S.-S., Ilyas M., Wu G., Yang F.-L. (2020). Downregulation of chitin synthase A gene by diallyl trisulfide, an active substance from garlic essential oil, inhibits oviposition and alters the morphology of adult *Sitotroga cerealella*. J. Pest Sci..

[14] Yan W., Wu M.-Y., Shah S., Yao Y.-C., Elgizawy K.K., Tang N., Wu G., Yang F.-L. (2021). Silencing the Triacylglycerol Lipase (TGL) Gene Decreases the Number of Apyrene Sperm and Inhibits Oviposition in *Sitotroga Cerealella*. Cell. Mol. Life Sci..

[15] Shah S., Zhang S.-S., Elgizawy K.K., Yan W.-H., Tang N., Wu G., Yang F.-L. (2022). Diallyl trisulfide reduced the reproductive capacity of male *Sitotroga cerealella* via the regulation of juvenile and ecdysone hormones. Ecotoxicol. Environ. Saf..

[16] De Los Santos C.G. (2015). Analysis of Transitions in Sperm Motility.

[17] Omar S.H., Al-Wabel N.A. (2010). Organosulfur compounds and possible mechanism of garlic in cancer. Saudi Pharm. J..

[18] Benavides G.A., Squadrito G.L., Mills R.W., Patel H.D., Isbell T.S., Patel R.P., Darley-Usmar V.M., Doeller J.E., Kraus D.W. (2007). Hydrogen sulfide mediates the vasoactivity of garlic. Proc. Natl. Acad. Sci. USA.

[19] Ying Y.Y. (2019). Regulation of Diallyl Trisulfide on Post-Mating Effect of *Sitotroga cerealella* at Sublethal Concentration. Master’s Thesis.

[20] Werner M., Simmons L.W. (2008). Insect Sperm Motility. Biol. Rev. Camb. Philos. Soc..

[21] Tremoen N.H., Gaustad A.H., Andersen-Ranberg I., van Son M., Zeremichael T.T., Frydenlund K., Grindflek E., Våge D.I., Myromslien F.D. (2018). Relationship between sperm motility characteristics and ATP concentrations, and association with fertility in two different pig breeds. Anim. Reprod. Sci..

[22] Toprak U., Hegedus D., Doğan C., Güney G. (2020). A journey into the world of insect lipid metabolism. Arch. Insect Biochem. Physiol..

[23] Frühbeck G., Méndez-Giménez L., Fernández-Formoso J.-A., Fernández S., Rodriguez A. (2014). Regulation of adipocyte lipolysis. Nutr. Res. Rev..

[24] Baer B., Eubel H., Taylor N.L., O’Toole N., Millar A.H. (2009). Insights into female sperm storage from the spermathecal fluid proteome of the honeybee *Apis mellifera*. Genome Biol..

[25] Prokupek A.M., Kachman S.D., Ladunga I., Harshman L.G. (2009). Transcriptional profiling of the sperm storage organs of *Drosophila melanogaster*. Insect Mol. Biol..

[26] Das C.C., Kaufmann B.P., Gay H. (1964). Histone-protein transition in *Drosophila melanogaster*: I. Changes during spermatogenesis. Exp. Cell Res..

[27] Srivastava V.K., Kumar K. (1984). Effect of the chemosterilant bisazir on the testes of the spotted bollworm *Earias fabia* Stoll. Toxicology.

[28] Paul S., Ghost M.R., Sahu C.R. (1991). Effects of insecticides on protein histochemistry of the testis of *Diacrisia obliqua* Walker (Lepidoptera: Arctiidae). Environ. Ecol..

