# Supplementary material for: Diallyl Trisulfide, a Biologically Active Component of Garlic Essential Oil, Decreases Male Fertility in Sitotroga cerealella by Impairing Dimorphic Spermatogenesis, Sperm Motility and Lipid Homeostasis
Source: Cells. 2023 Feb 20;12(4):669. doi: 10.3390/cells12040669 (PMC9955147; doi:10.3390/cells12040669)
Supplement: Supplementary file 1 [file cells-12-00669-s001.zip › cells-2120305-Supplementary Captions.pdf]

## Supplementary Captions

**Movie S1.** Representative image of sperm videography of phase contrast microscopy of control (CK) samples. Female reproductive tracts were dissected from mated female upon mating completion. The sperm were analyzed under the phase contrast microscope at a rate of 17 frames per second. The videos were further analyzed with ImageJ for different parameters measurements.

**Movie S2.** Representative image of sperm videography of phase contrast microscopy of DAT fumigated samples. Female reproductive tracts were dissected from mated female upon mating completion. The sperm were analyzed under the phase contrast microscope at a rate of 17 frames per second. The videos were further analyzed with ImageJ for different parameters measurements.
